# Supplementary material for: On the molecular origins of the ferroelectric splay nematic phase
Source: Nat Commun. 2021 Aug 16;12:4962. doi: 10.1038/s41467-021-25231-0 (PMC8367997; doi:10.1038/s41467-021-25231-0)
Supplement: Supplementary file 1 — Supplementary Information [file 41467_2021_25231_MOESM1_ESM.pdf]

Supplementary Information

On the molecular origins of the ferroelectric splay nematic phase

Richard J. Mandle[1,2], Nerea Sebastián[3], Josu Martinez-Perdiguero[4] & Alenka Mertelj[3]

[1] School of Physics and Astronomy, University of Leeds, Leeds, UK, LS2 9JT
[2] Department of Chemistry, University of York, York, YO10 5DD, UK
[3] Jožef Stefan Institute, SI-1000 Ljubljana, Slovenia
[4] Department of Physics, University of the Basque Country (UPV/EHU), Apdo.644-48080 Bilbao, Spain

Supplementary Note 1 - Phase behaviour as observed by Polarizing Optical Microscopy

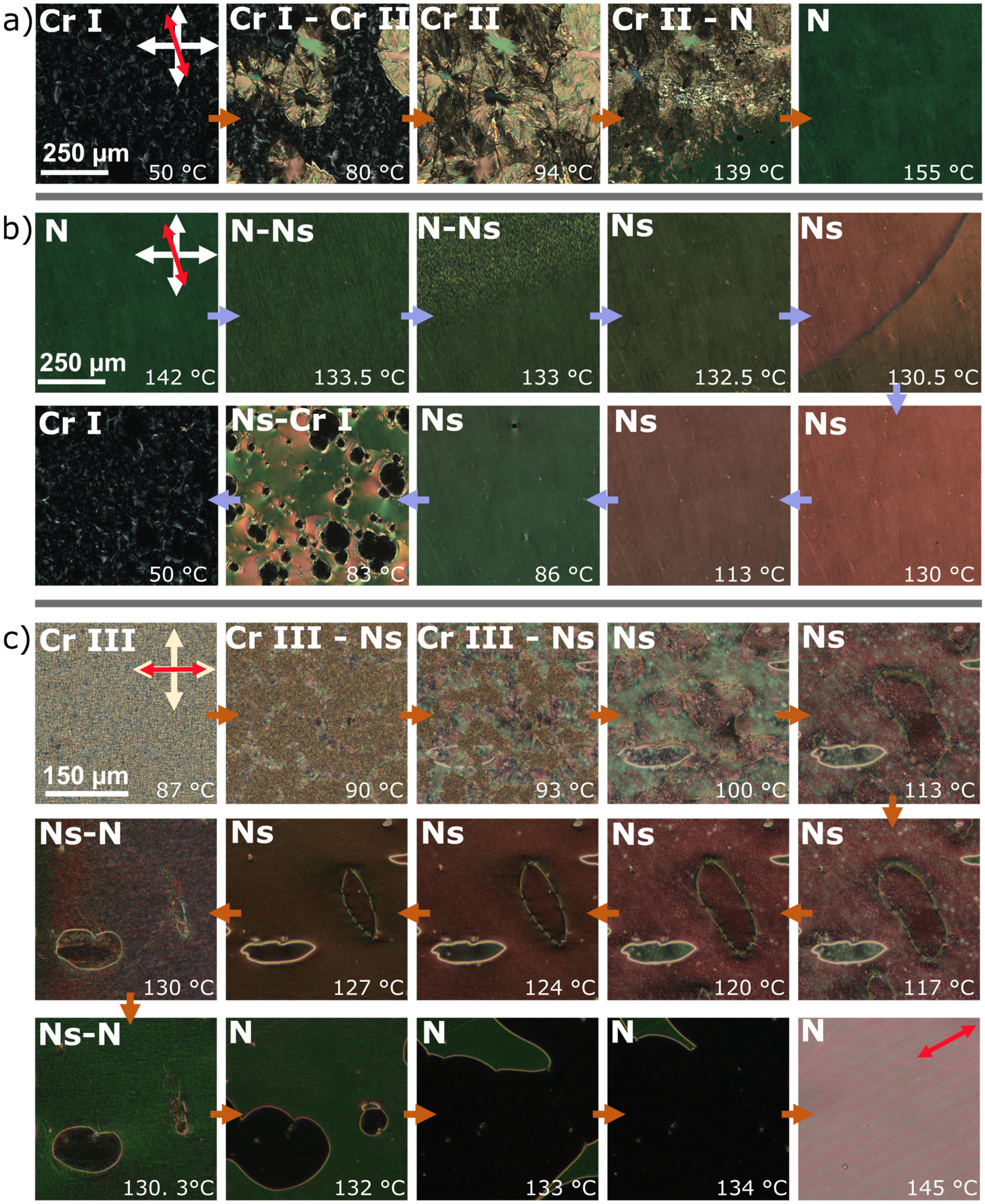

Supplementary Fig. 1: Phase behaviour of RM734 as investigated by polarizing optical microscopy. (a) on heating at 5 °C/min from room temperature. (b) On cooling from the N phase down to room temperature at 2 °C/min. (c) Under certain conditions a third crystalline phase is obtained when cooling from the Ns phase. On subsequent heating, Cr-III melts directly into the Ns phase and Ns-N transition is observed around 130 °C. Domain structures shown in (c) are discussed elsewhere.[1]

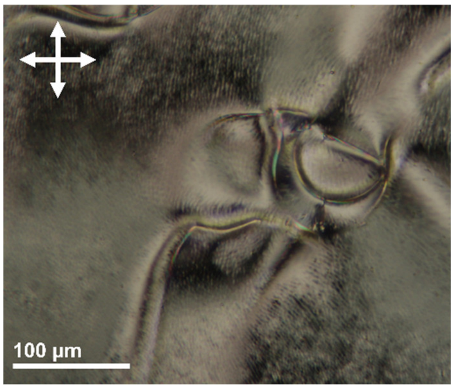

Supplementary Fig. 2: Polarizing optical microscopy image of RM734 at room temperature after fast cooling from the N phase, showing that under certain conditions N_S phase can be supercooled down to room temperature.

Supplementary Note 2 - Dielectric Spectroscopy RM734-CN

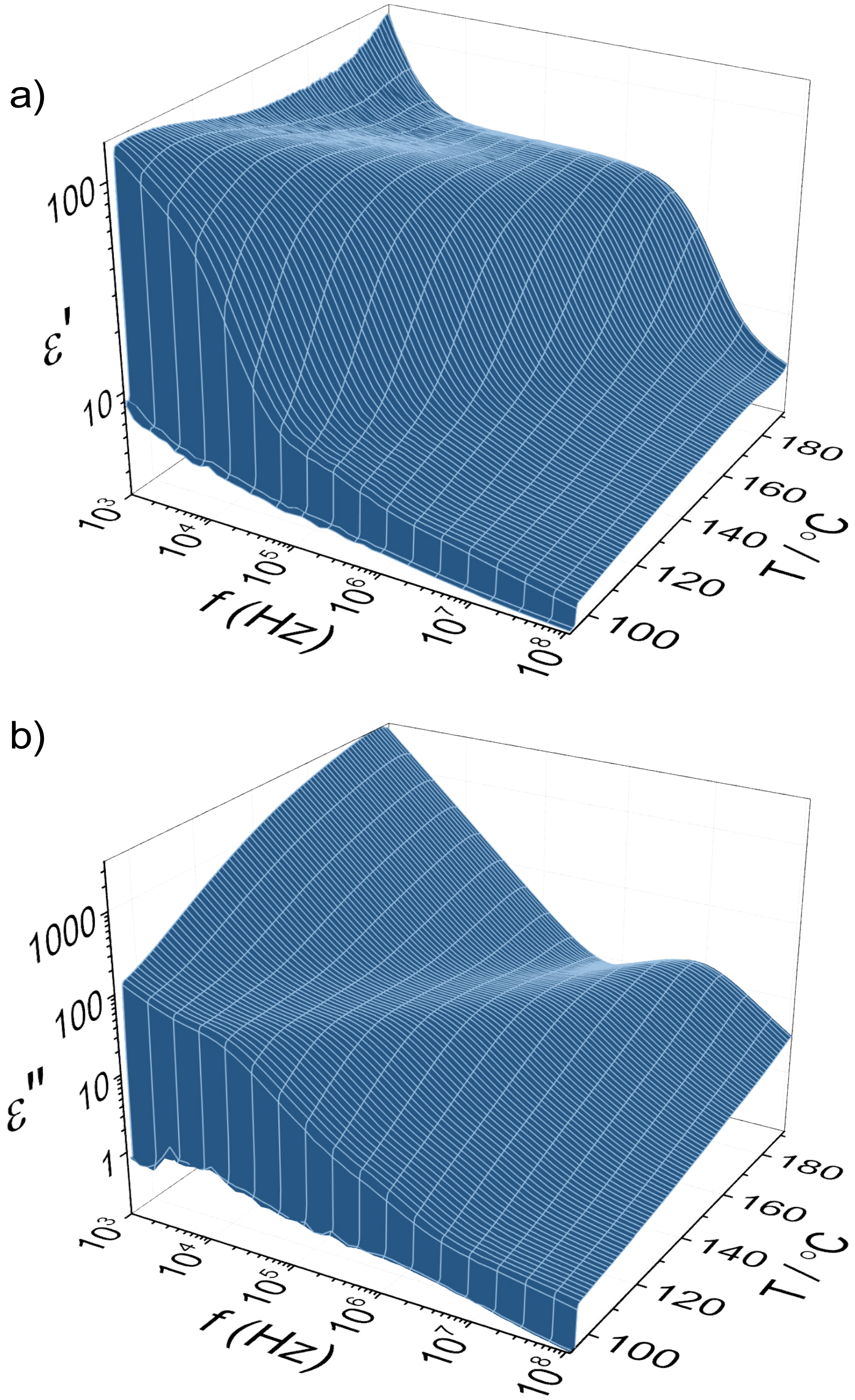

Supplementary Fig. 3: Dielectric spectra of RM734-CN in the N phase. Temperature and frequency dependence of the (a) real and (b) imaginary components of the dielectric spectra of RM734-CN.

To obtain the frequency and amplitude of each relaxation process a simultaneous fitting of the real and imaginary parts of $\varepsilon(\omega)$ was performed at each temperature employing the Havriliak-Negami processes[2]:

$$\varepsilon(\omega) = \sum_k \frac{\Delta\varepsilon_k}{[1 + (i\omega\tau_{HN})^{\alpha_k}]^{\beta_k}} + \varepsilon_\infty - \frac{\sigma_0}{\omega\varepsilon_0} \quad (1)$$

The best fit parameters are shown in Fig. 2 of the main text together with two plots at different temperatures of the derivative of $\varepsilon(\omega)$ showing the good agreement of the obtained fits. Although the fit was performed directly on $\varepsilon(\omega)$, the reason for using the derivative of the real part in the shown plots is that it allows for much better visualization of the deconvolution of the data into the different relaxations. It is worth mentioning here that the discontinuity at 172 °C in the RM734 data of main manuscript Fig. 2b-c is solely due to the impossibility of uniquely deconvoluting the $\mathrm{m}_{\parallel,1}$ and $\mathrm{m}_{\parallel,2}$ modes above that temperature during the data-fitting process. The modes heavily overlap in the frequency spectra and resolving them in a robust way without making unsubstantiated assumptions becomes only feasible below that temperature. At higher temperatures we only used one broad mode for the fit (red circles in Fig. 2 between 172 °C and 200 °C) but we imply that both modes are present up to the isotropic phase however we are not able to separately deconvolute them unambiguously.

In the high range of the measured frequencies (1-10 MHz) a third mode is detected, $\mathrm{m}_{\parallel,3}$. At the I-N transition, its frequency is larger than that of $\mathrm{m}_{\mathrm{iso}}$ and rapidly climbs out of the measured frequency range. On further cooling, its characteristic frequency decreases and the mode is again detected. This mode, by frequency and amplitude, can be associated with the rotation around the molecular long axis, as described by the Nordio-Rigatti-Segre theory[3].

The same reasoning applies to the previously reported RM734 data[4], which shows the 'splitting' in two modes below 160 °C and a high frequency mode appearing in the measured range at around that same temperature.

For completeness, Supplementary Fig. 4 and Supplementary Fig. 5 show more fit examples for RM734-CN and RM734 respectively, including temperatures close to the above-mentioned 'splitting' points.

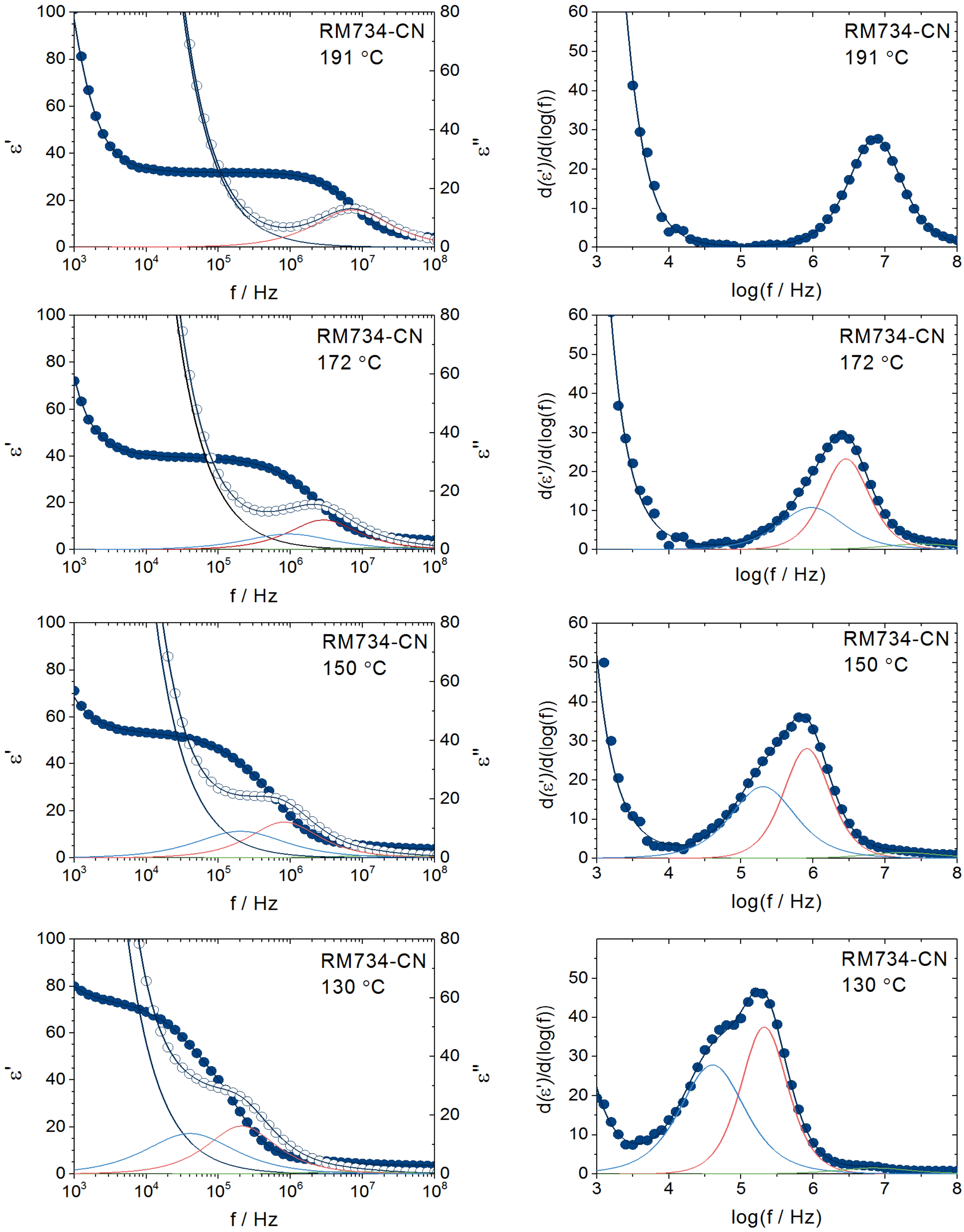

Supplementary Fig. 4: Examples at different temperatures of the measured dielectric spectra and their fits for RM734-CN. (left) Frequency dependence of the real (full circles) and imaginary (open circles) dielectric permittivity. Solid lines result from fitting to Supplementary Equation 1 and the corresponding deconvolution into the elementary processes. Dashed lines correspond to the current conductivity term. (right) The derivative of the real part of the permittivity $\mathrm{d}(\varepsilon')/\mathrm{d}\log(f)$ at the corresponding temperatures allows for better visualization of the relaxation modes and benevolence of the fits. Modes $\mathrm{m}_{\parallel,1}$ and $\mathrm{m}_{\parallel,2}$ overlap in frequency at high temperatures and is not possible to resolve them above 160 °C. For those high temperatures, only one mode was considered in the fitting.

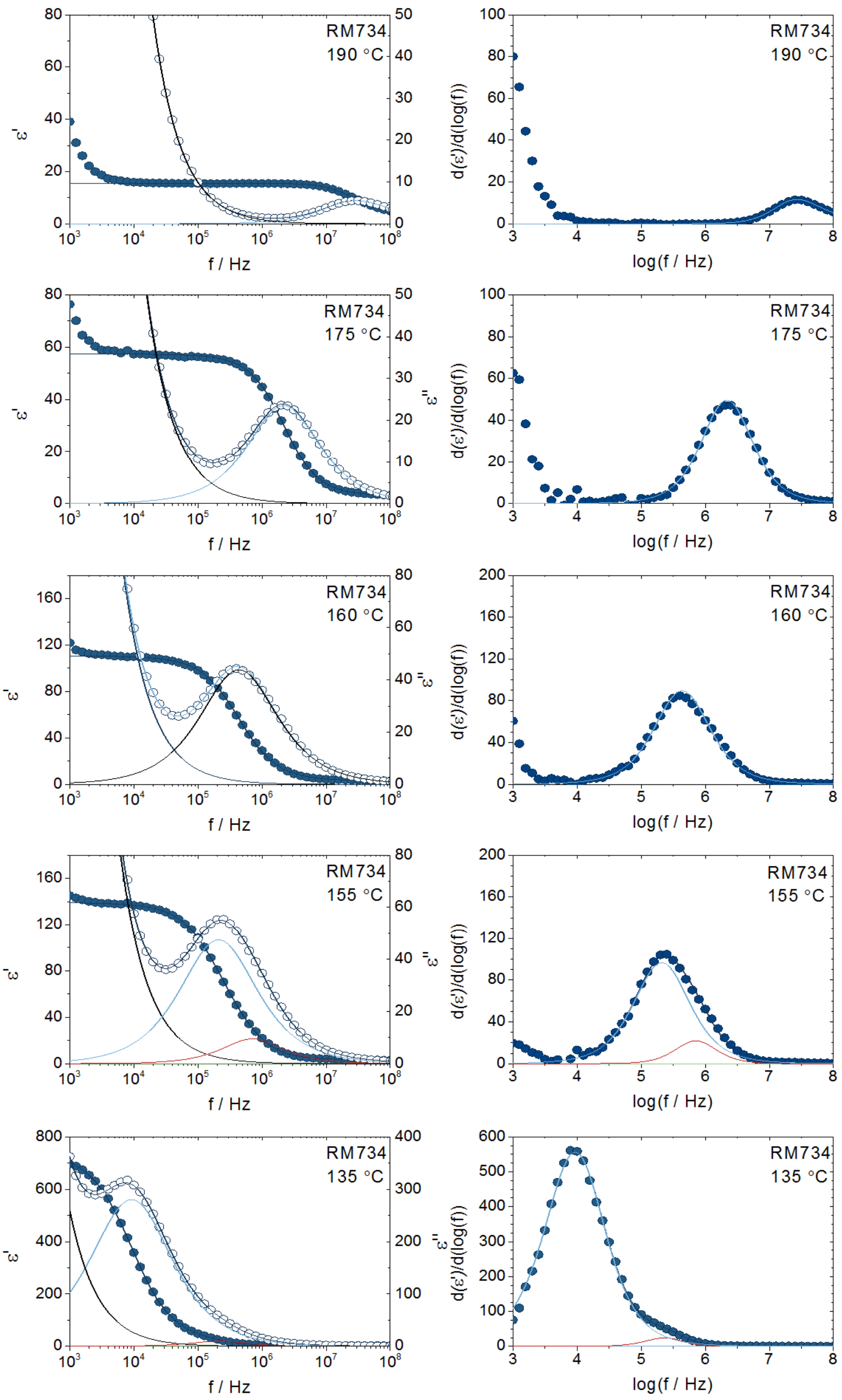

Supplementary Fig. 5: Examples at different temperatures of the measured dielectric spectra and their fits for RM734. (left) Frequency dependence of the real (full circles) and imaginary (open circles) dielectric permittivity. Solid lines result from fitting to Supplementary Equation 1 and the corresponding deconvolution into the elementary processes. Dashed lines correspond to the current conductivity term. (right) The derivative of the real part of the permittivity $\mathrm{d}(\varepsilon')/\mathrm{dlog}(f)$ at the corresponding temperatures allows for better visualization of the relaxation modes and benevolence of the fits. Modes $\mathrm{m}_{\parallel,1}$ and $\mathrm{m}_{\parallel,2}$overlap in frequency at high temperatures and is not possible to resolve them above 160 °C. For those high temperatures only one mode was considered in the fitting, where the amplitude of the lower frequency mode $\mathrm{m}_{\parallel,1}$makes it prevail. Source of data and fits: 4.

Supplementary Note 3 - Birefringence and order parameter

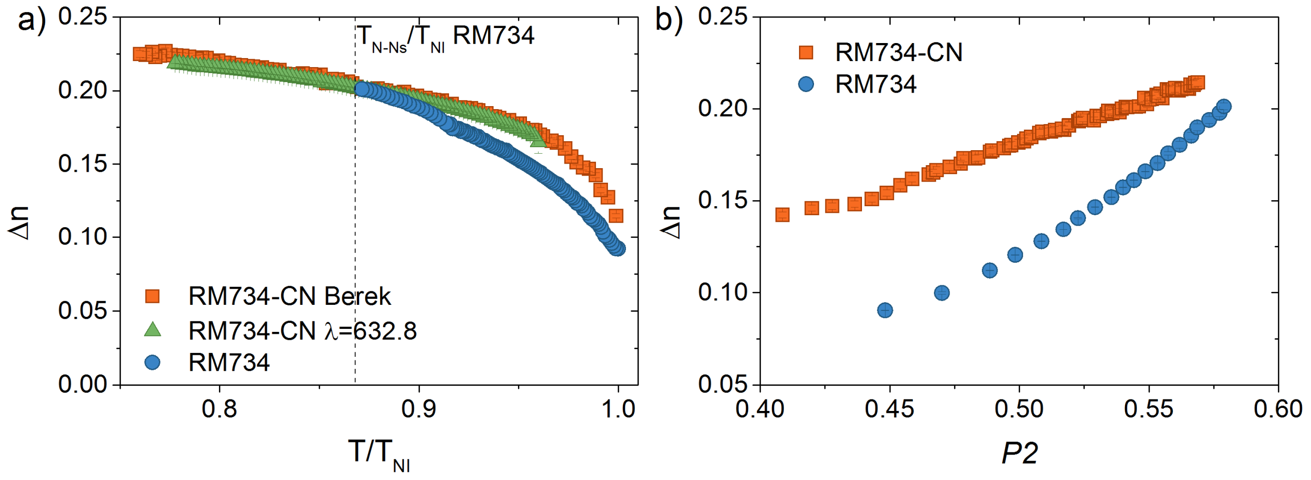

Supplementary Fig. 6: Birefringence measurements. a) Comparison of the temperature dependence of the birefringence of RM734-CN and RM734. The anisotropy of the index of refraction Δn of RM734-CN was measured by polarization microscopy. A d=20 μm cell with planar alignment (director in the cell plane) was placed between crossed polarizers with the director at 45 degrees with respect to them. The phase difference between the ordinary and the extraordinary light $\phi = 2\pi\Delta nd/\lambda$ was calculated from the intensity of monochromatic light ($\lambda = 632.8$ nm) transmitted through the sample. Additionally, the temperature dependence of Δn was measured with a Berek compensator in a 9 μm cell. As shown by the figure both results are comparable. Results for RM734-CN are also compared to Δn of RM734 [5] (b) Representation of the birefringence vs experimental values of *P2* [6]. Given the comparable polarizabilities of both materials, the plot reflects the difference in molecular orientational correlations in the N phase of RM734 and RM734-CN.

Supplementary Note 4 - Fredericks transition RM734-CN

A reference value for the splay elastic constant was measured from the change in the dielectric permittivity when a variable voltage is applied to a planar aligned sample. The frequency was set to 30 kHz to avoid undesired ionic effects and the cell's ITO relaxation. Experimental results were fitted to equations:

$$V = \frac{2V_{th}}{\pi}\int_{\psi_0}^{\frac{\pi}{2}}\left[\frac{1+\kappa\eta\sin^2\psi}{(1+\gamma\eta\sin^2\psi)(1-\eta\sin^2\psi)}\right]^{\frac{1}{2}}\mathrm{d}\psi \tag{2}$$

$$C = C_{\perp}\frac{\int_{\psi_0}^{\frac{\pi}{2}}\left[\frac{(1+\gamma\eta\sin^2\psi)(1+\kappa\eta\sin^2\psi)}{(1-\eta\sin^2\psi)}\right]^{\frac{1}{2}}\mathrm{d}\psi}{\int_{\psi_0}^{\frac{\pi}{2}}\left[\frac{(1+\kappa\eta\sin^2\psi)}{(1+\gamma\eta\sin^2\psi)(1-\eta\sin^2\psi)}\right]^{\frac{1}{2}}\mathrm{d}\psi} \tag{3}$$

Where the parameter η is related to the maximum tilt angle at the centre of the cell ϕ_m $(\eta = \sin^2(\phi_m))$; the parameters γ and κ correspond to the reduced quantities $\gamma = \varepsilon_{\perp}/\varepsilon_{\parallel} - 1$ and $\kappa = K_3/K_1 - 1$. $\varepsilon_{\perp}$corresponds to the value of the permittivity below the threshold voltage (V_{th}) for the Fredericksz transition. $\varepsilon_{\parallel}$ corresponds to the dielectric permittivity values for saturated director reorientation. Values of V_{th} and $\Delta\varepsilon$ are then used to calculate the splay elastic constant $K_1 = (V_{th}/\pi)^2\varepsilon_0\Delta\varepsilon$.

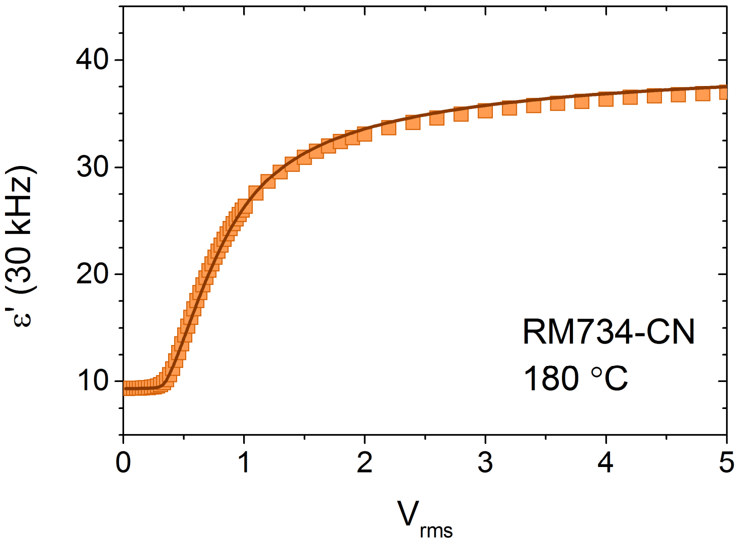

Supplementary Fig. 7: Fredericks transition. Voltage dependence of the real permittivity at 30 kHz at 180 °C. Solid line shows the fit to Supplementary Equation 2 and 3.

Supplementary Note 5 - Polarizabilities, inertia tensor and dipole direction of *RM734* and *RM734-CN*

The polarizability tensors (Supplementary Table 1) of RM734 and RM734-CN were calculated at the M06HF-D3/aug-cc-pVDZ level of DFT for a wavelength of 800 nm.[7–10]

	λ (nm)	Iso ($C^2m^2J^{-1}$)	Aniso ($C^2m^2J^{-1}$)	Eigenvalues of the static polarizability tensor ($C^2m^2J^{-1}$)
RM734	800	44.7	45.7	{75.0844, 43.3846, 19.6424}
RM734-CN	800	46.4	50.8	{77.7493, 41.9472, 19.5296}

Supplementary Table 1: Polarizabilities of RM734 and RM734-CN as calculated using M06HF-D3/aug-cc-pVDZ level of DFT.

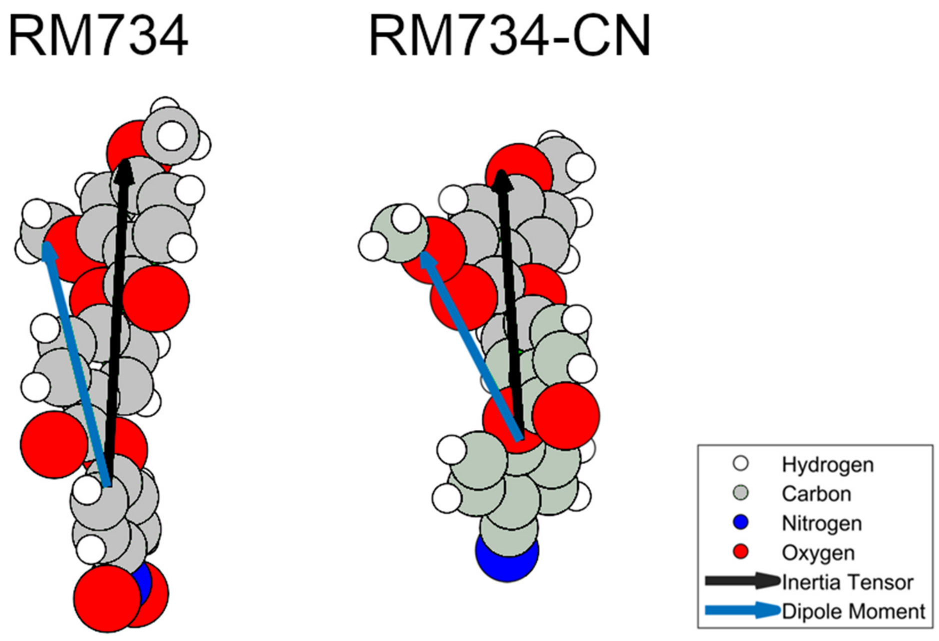

Supplementary Fig. 8: Directions of the mass inertia axis and dipole moment vector for RM734 and RM734-CN as calculated using M06HF-D3/aug-cc-pVTZ level of DFT**.**

Suplementary Note 6 - Conformational distributions calculated from MD simulations

We extracted the conformational distributions of several dihedrals from MD simulations of RM734 and RM734-CN in both polar and apolar configurations at simulation temperatures of 400K as a means to complement torsional potentials calculated at the DFT(M06HF-D3/aug-cc-pVTZ) level which are discussed in the manuscript. Dihedral angles were calculated from the atomic coordinates over the full production MD trajectory and were binned into histograms to give the plots shown below. Differences in conformational populations between the two materials and two configurations are minor.

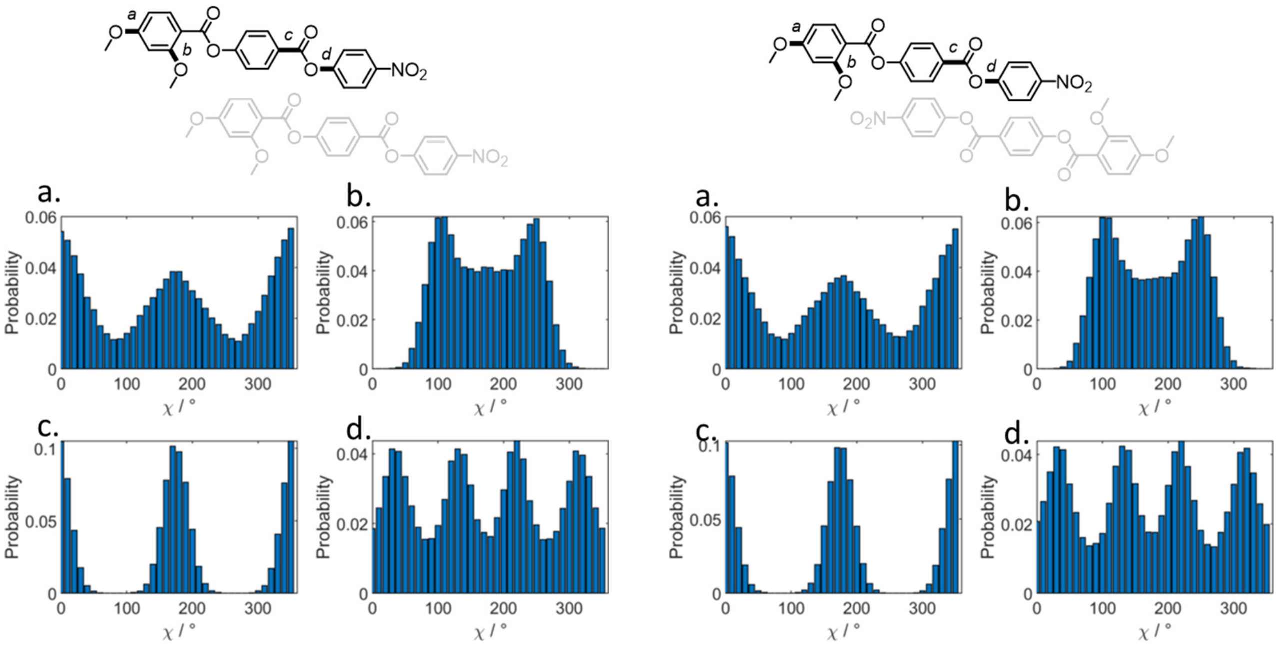

Supplementary Fig. 9: Conformational distributions from an MD simulation of RM734 at 400K in the (left) polar nematic configuration and (right) apolar nematic configuration.

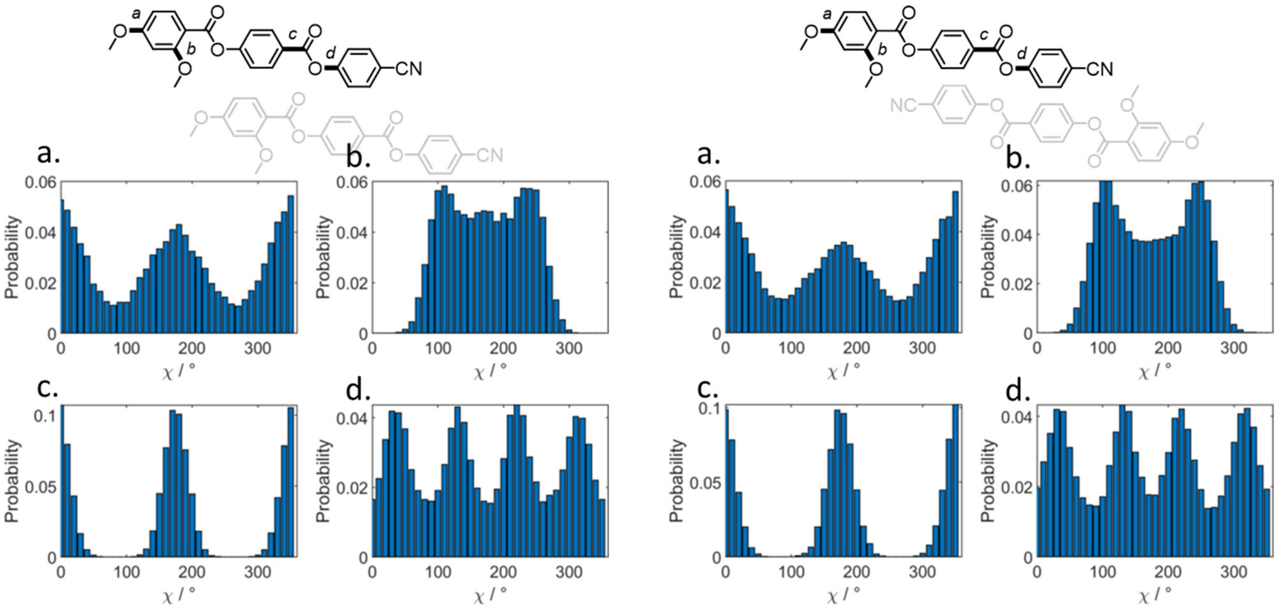

Supplementary Fig. 10: Conformational distributions from an MD simulation of RM734-CN at 400K in the (left) polar nematic configuration and (right) apolar nematic configuration.

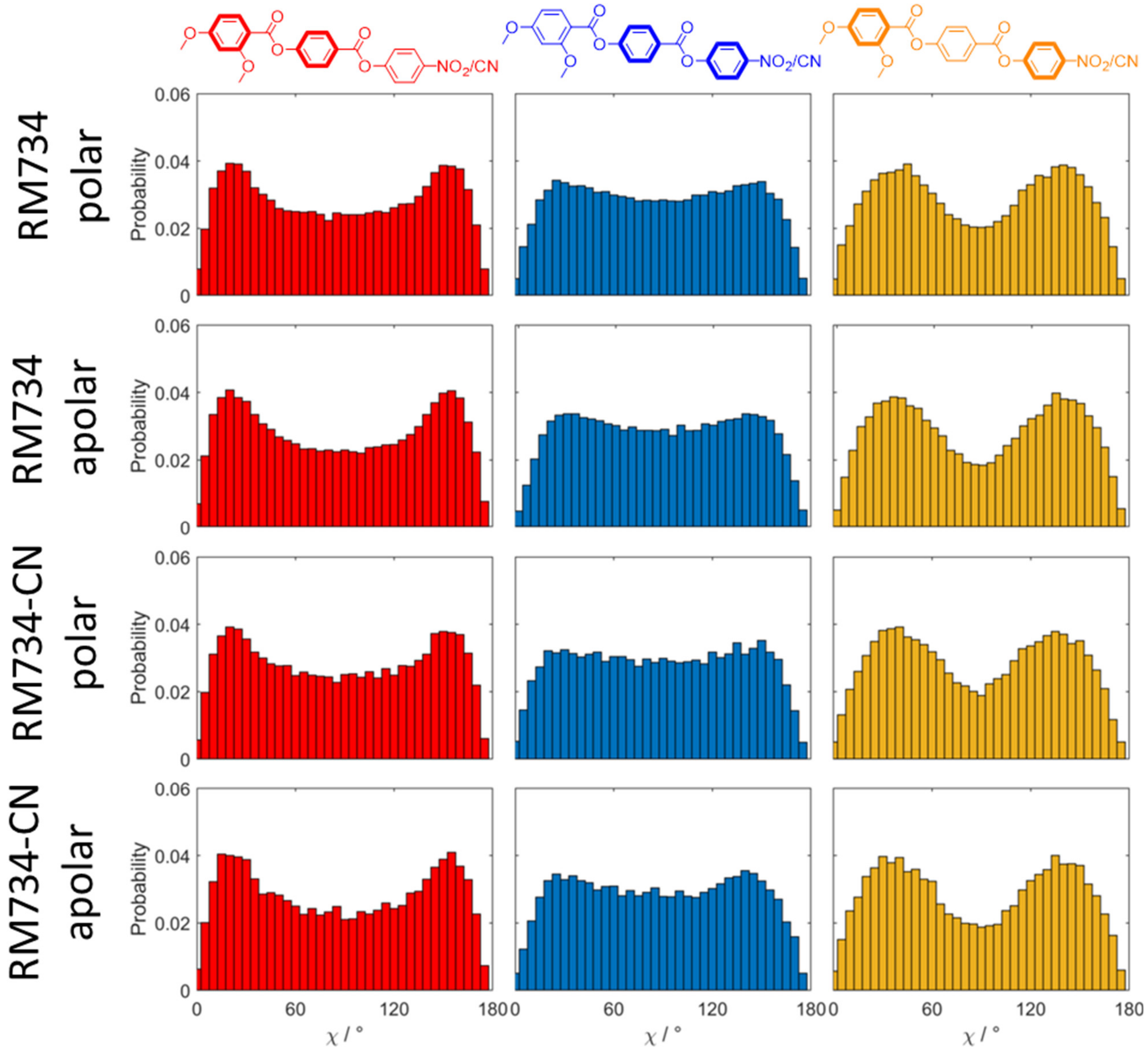

Supplementary Fig. 11: Angular distribution between the planes of the aromatic rings at 400K of RM734 and RM734-CN in the polar and apolar configuration.

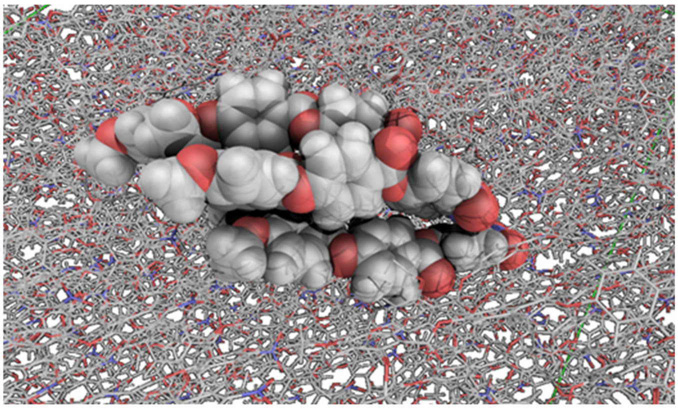

Supplementary Fig. 12: Visualization of staggered positions for RM734 molecules in the polar configuration as represented from MD trajectories.

Supplementary Note 7 – Pair correlation functions calculated from MD

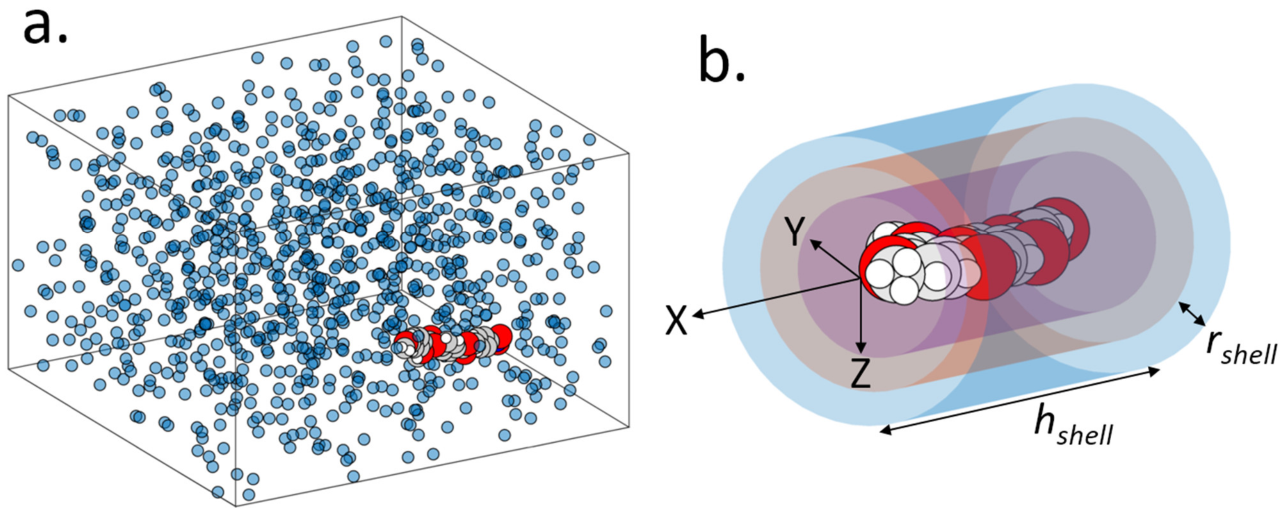

Supplementary Fig. 13: An instantaneous configuration of RM734 in the polar nematic configuration with a single molecule shown as space-filling model, and the molecular centre of mass shown for all remaining molecules; (b) Illustration of the cylindrical averaging process for a single molecule oriented with its mass inertia axis along X.

Supplementary Note 8 – Radial distribution functions

RDFs computed between atoms in the nitro (NO_2) or cyano (CN) groups and either the closest carboxylate ester (C(O)O) or terminal methoxy (OCH_3) groups. RDFs were computed over the entire production MD trajectory (250 ns total) for RM734 and RM734-CN respectively, in either the polar or apolar configurations. The intermolecular RDF between given sets of atoms is presented as a solid line, whereas the dashed line corresponds to the intramolecular RDF for the same set of atoms.

The RDF presented here are isotropic, being calculated for spherical shells, and are calculated between specific sets of atoms corresponding to functional groups within a given molecule. On the other hand, the pair correlation functions (PCF) presented within the manuscript are anisotropic, being calculated for cylindrical shells oriented with their length along the nematic director, and are computed between the centres-of-mass of all molecules within the simulation.

Representative configurations were selected using selection algebra tools within Pymol; pairs were chosen with a distance between selected group atoms chosen to reproduce a given peak in the RDF. A random pair was picked from a randomly chosen trajectory timestep, the image of the pair was raytraced and the average distance between group atoms was computed.

We link the calculated pairs with the RDF plots using the coloured arrows shown below in Supplementary Fig. 14 and 15.

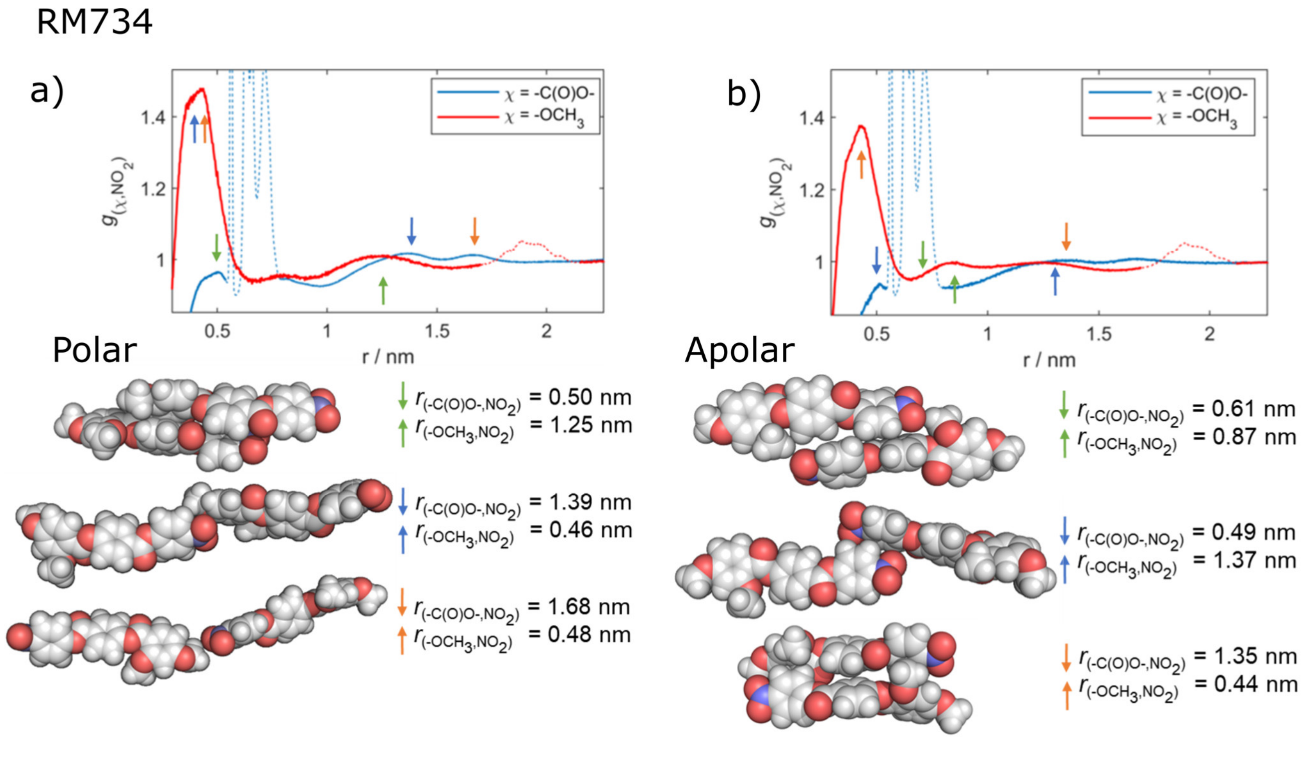

Supplementary Fig. 14: RDF for RM734 in the (a) polar configuration and (b) apolar configuration computed between the nitro group and proximal carboxylate ester (blue) or terminal methoxy group (red). The intermolecular RDF between given sets of atoms is presented as a solid line, whereas the dashed line corresponds to the intramolecular RDF for the same set of atoms. Representative configurations shown below, along with relevant average distances between group atoms.

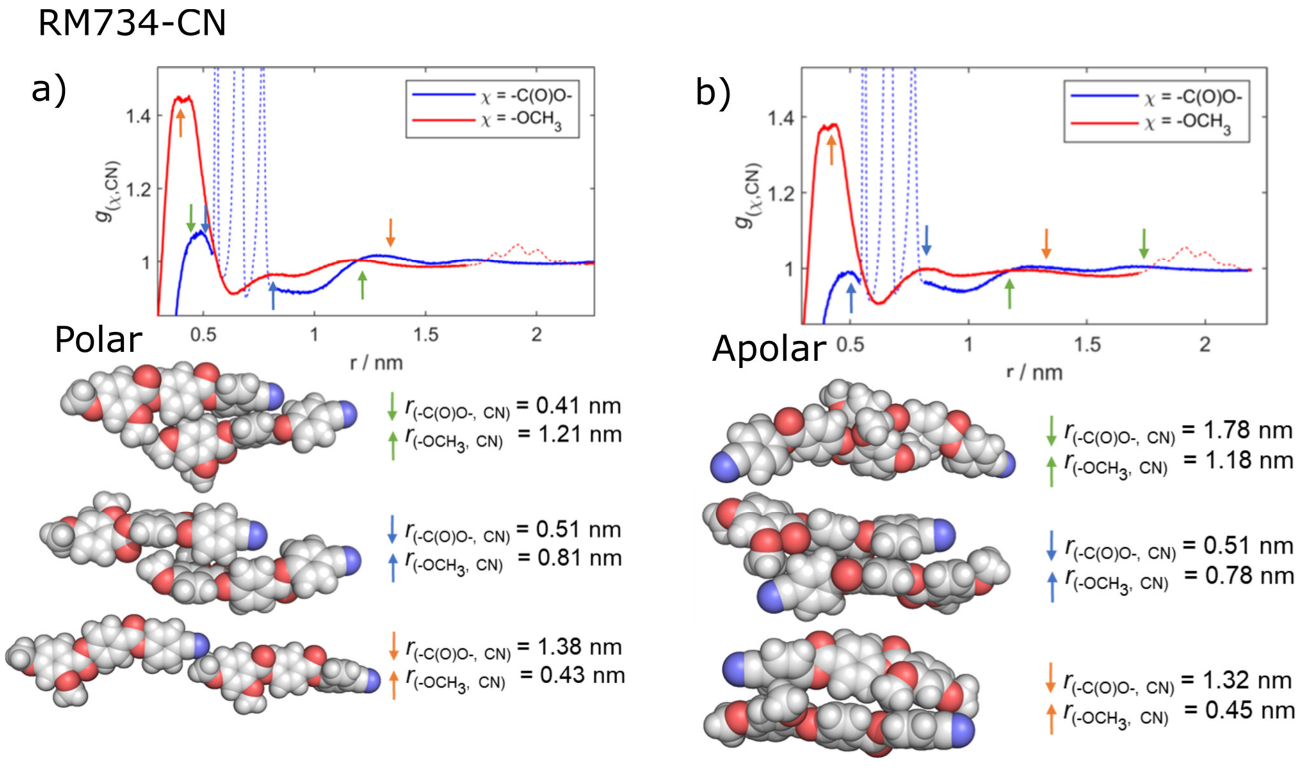

Supplementary Fig. 15: RDF for RM734-CN in the (a) polar configuration and (b) apolar configuration computed between the nitro group and proximal carboxylate ester (blue) or terminal methoxy group (red). The intermolecular RDF between given sets of atoms is presented as a solid line, whereas the dashed line corresponds to the intramolecular RDF for the same set of atoms. Representative configurations shown below, along with relevant average distances between group atoms.

Supplementary Note 9 - Experimental and calculated scattered intensities for RM734

For each MD simulation, we calculated two-dimensional WAXS patterns as an average of trajectories in the time window 200 – 280 ns, as described in the experimental section of the manuscript. For the resulting 2D WAXS patterns, we then calculated orientational order parameters as described elsewhere. Although this is an unconventional way to obtain orientational order parameters from MD simulations, it makes the same assumptions as used for experimental data and so provides directly comparable results. Reassuringly, values obtained from simulated WAXS patterns are not significantly different from the values obtained directly from MD simulations at the same temperature; for RM734 we obtain *P2* values of 0.78 and 0.73 in the polar and apolar states, respectively, and for RM734-CN we obtain *P2* values of 0.75 and 0.74 in the polar and apolar states, respectively.

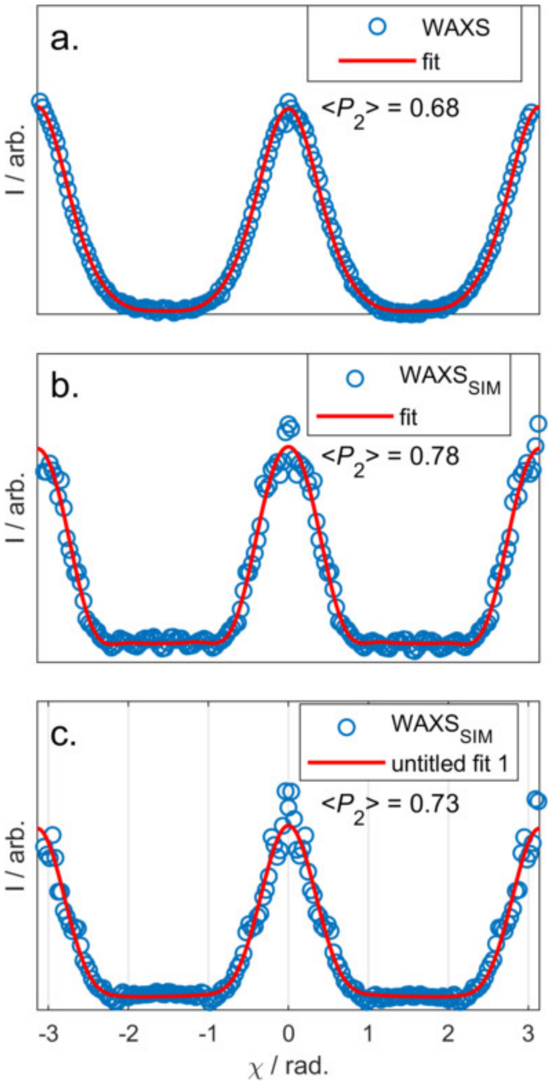

Supplementary Fig. 16: Scattered intensity as a function of χ for radially integrated wide-angle X-ray scattering patterns of RM734: (a) experimental data from reference [6]; simulated data for the polar (b) and apolar (c) nematic configurations, obtained by azimuthal integration of simulated 2D WAXS patterns in the Q range 1 – 1.6 Å^{-1}. In all three examples data were fitted to

$I(X) = \sum_0^{\infty} \frac{\pi}{2} f_{2n} \frac{(2n-1)!!}{2^n n!} cos^{2n} X$, and $<P_2>$ calculated as $\langle P_2 \rangle = \frac{1}{2}(3\langle cos^2\beta \rangle - 1)$, where

$\langle cos^2\beta \rangle = \sum_{n=0}^{\infty} \frac{f_{2n}}{2n+3} \Big/ \sum_{n=0}^{\infty} \frac{f_{2n}}{2n+1}$

Supplementary note 10 - Energy per molecule as calculated from MD

Energy per molecule is calculated from the total simulation energy (average over the whole production MD trajectory) and divided by the number of molecules. One should keep in mind that numbers are approximate and typically, only relative changes are meaningful. Consequently, comparisons between materials should be avoided. Relative energy difference between the polar and the apolar configurations shows that for RM734 polar configuration is more favourable. In the case of RM734-CN, the difference between both configurations is negligible.

	Configuration	E Total (kJ/mol)	E molecule (kJ/mol)	ΔE molecule (kJ/mol)
RM734	POLAR	-53189.6	-78.22	2.19
	APOLAR	-51700.4	-76.03	
RM734-CN	POLAR	-371396	-546.17	0.14
	APOLAR	-371304	-546.03	

Supplementary Table 2: MD simulation total energies and energy per molecule at 400K of *RM734* and *RM734-CN*.

Supplementary note 11 – Density and molecular diffusion coefficients as calculated from MD

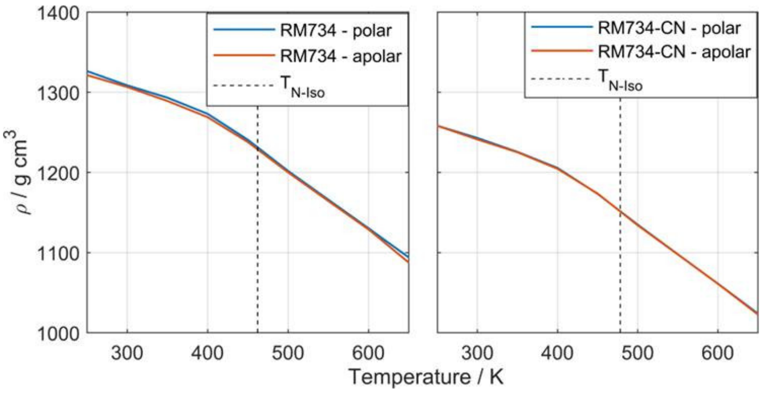

Supplementary Fig. 17: Plots of the mean calculated simulation density as a function of simulation temperature for RM734 and RM734-CN in polar and apolar configurations.

Molecular diffusion coefficients were calculated from the MD simulation trajectories for RM734 and RM734-CN in both polar and apolar states using the Gromacs tool gmx msd as supplied with Gromacs/2019.2.

	Configuration	D m^2/s
RM734	POLAR	4.3±0.6 10^{-12}
	APOLAR	1.1±0.4 10^{-12}
RM734-CN	POLAR	3.63±0.08 10^{-12}
	APOLAR	4.7±0.2 10^{-12}

Supplementary Table 3: Calculated translational diffusion constant *D* for RM734 and RM734-CN MD simulations at 400 K

Supplementary Note 12 - RM554

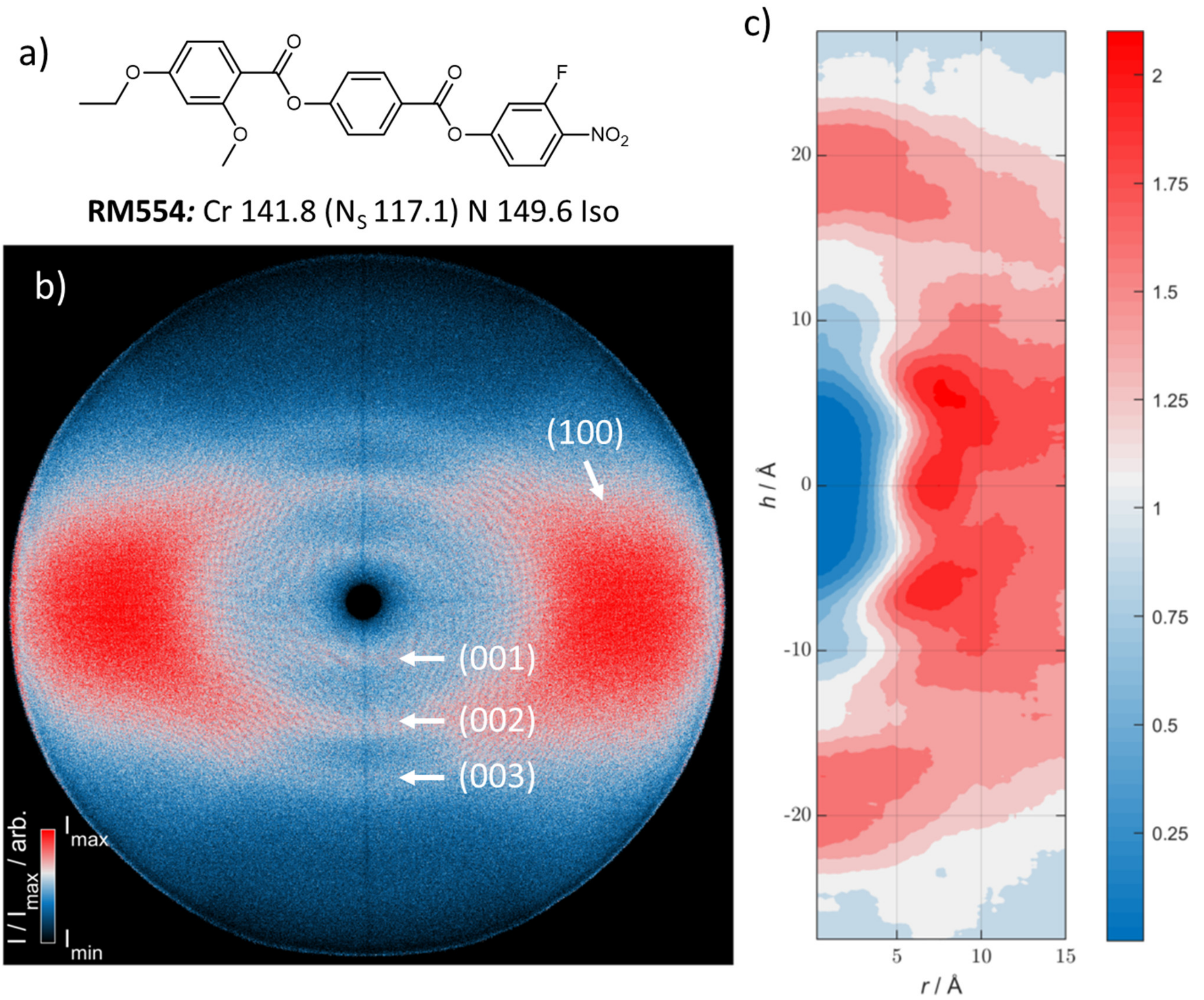

Supplementary Fig. 18: RM554. (a) Molecular structures and transition temperatures (°C), (b) Magnetically aligned two dimensional X-ray scattering patterns in the N_S phase at 87 °C. X-ray pattern shows the wide angle (100) and low angle (001) scattering peaks characteristic of classical nematic materials and exhibits additional diffuse small angle reflections (002) and (003) similarly to RM734. (c) cylindrical pair correlation function obtained for an MD simulations of RM554 in the polar nematic configuration at 400 K.

Supplementary Note 13 - Solid State of RM734

We revisited the solid-state crystal structure of RM734, which is available from the CCDC as deposition number 1851381. We find that, in agreement with DFT calculations for parallel pairs of RM734 (and also RM554, above), there is a 'close contact' between the carbon atom of the ester (COO) group and oxygen atom of the nitro (NO_2) group, with a distance of 3.33 Å (Supplementary Fig. 19). This distance is about 0.1 Å larger than the sum of the VDW radii of the atoms.

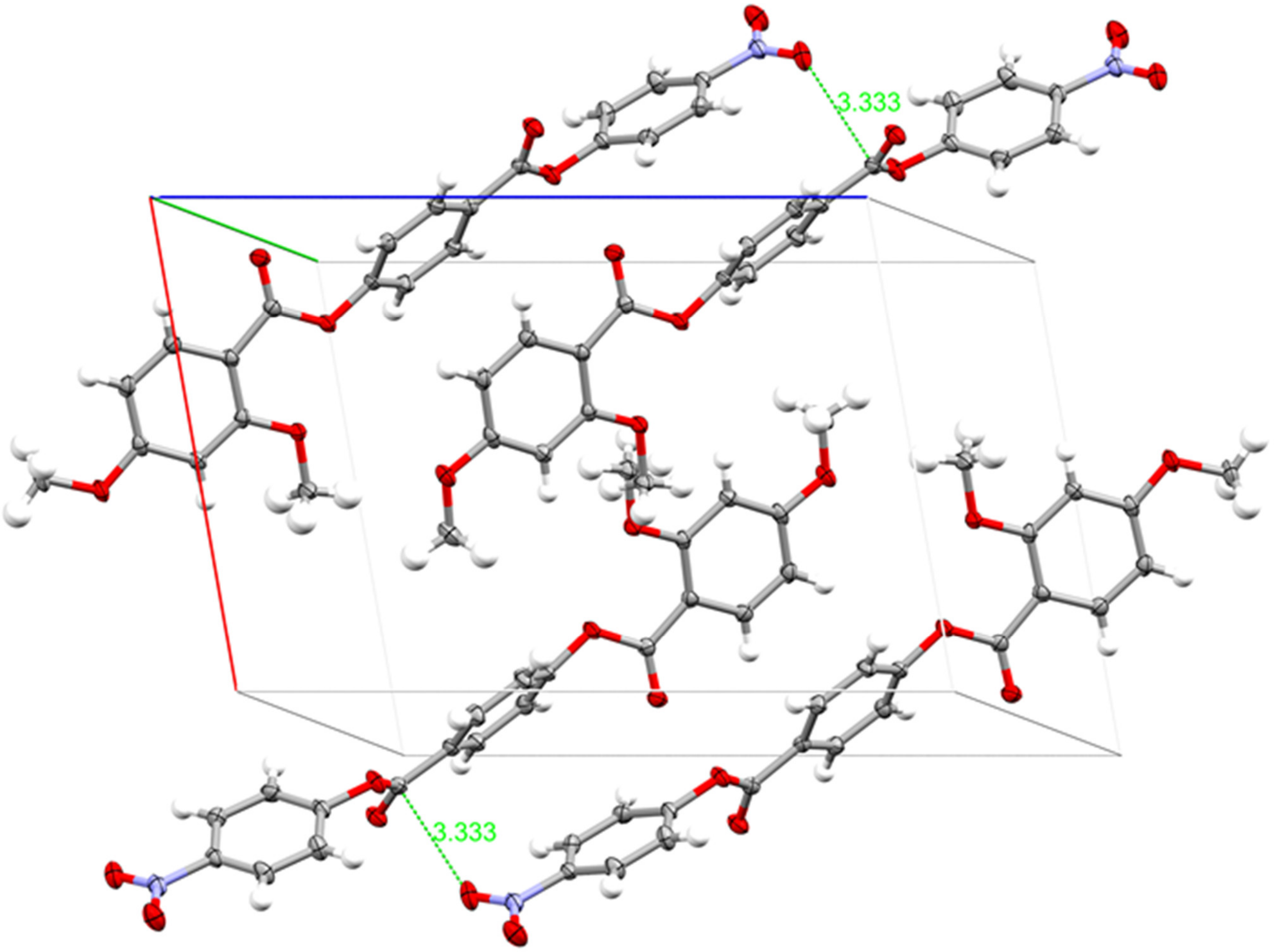

Supplementary Fig. 19: Structure of RM734 displayed as a thermal ellipsoid model (50% probability), obtained via X-ray diffraction. The unit cell (space group $P\overline{1}$) is indicated. Green lines correspond to nitro-ester close contacts, as described in the text. Viewed perpendicular to the AC plane, along the reciprocal B axis.

Supplementary Note 14 - RESP Charge Assignment

RESP charges were calculated at the B3LYP/6-31G(d,p) level of DFT using the Gaussian G09.d01 software package. First, we optimised geometry at the same level of DFT: [7–10]

#p opt b3lyp/6-31G(d,p) nosymm iop(6/7=3) gfinput

The RESP charges were then calculated for the optimised geometry by first computing the ESP charge in Gaussian G09.d01:

#p b3lyp/6-31G(d,p) nosymm iop(6/33=2) pop(chelpg,regular)

And then processing the output with the antechamber program in AmberTools 16.

We give charges for RM734 and RM734CN in Supplementary Fig. 20, below. We derived charges for other materials studied by molecular dynamics in the same way.

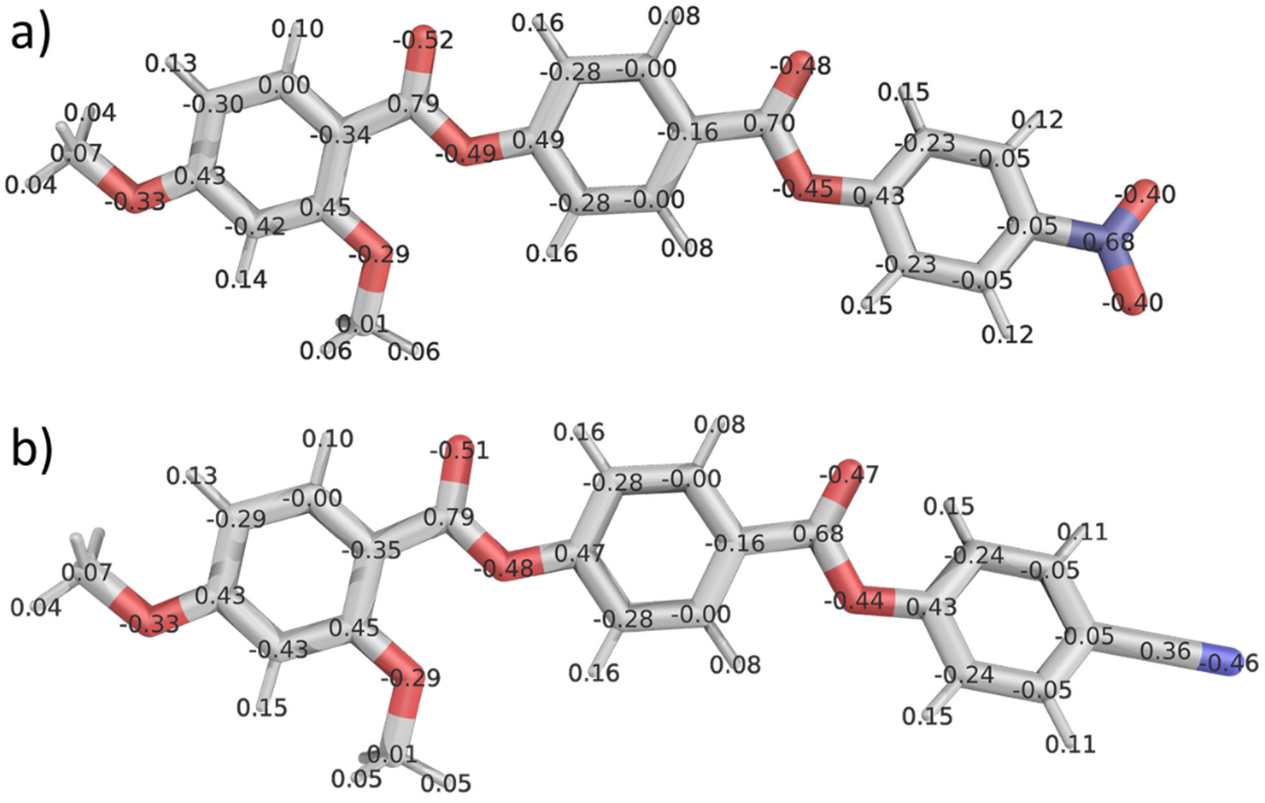

Supplementary Fig. 20: RESP charges calculated at the B3LYP/6-31G(d,p) level of DFT for (a) *RM734* and (b) *RM734CN*.

Supplementary Note 15 - Stability of Polar Order in MD Simulations

The temperature evolution of the order parameter <*P1*> can be used to confirm the thermodynamic stability of the polar and apolar nematic configurations; if the polar order is unstable <*P1*> will decay towards zero as the simulation progresses, whereas if polar order were favoured – for example by the application of a large potential - then in the apolar case <*P1*> will rise towards unity. As shown in Supplementary Fig. 21, the stable value of <*P1*> indicates that head-tail flipping of molecules is largely arrested, with little deviation from the mean value. As shown in the manuscript, when nematic simulations are heated into the isotropic liquid <*P1*> becomes near-zero, demonstrating that the lack of flipping is a phenomenon associated with nematic order rather than the specific molecules studied herein.

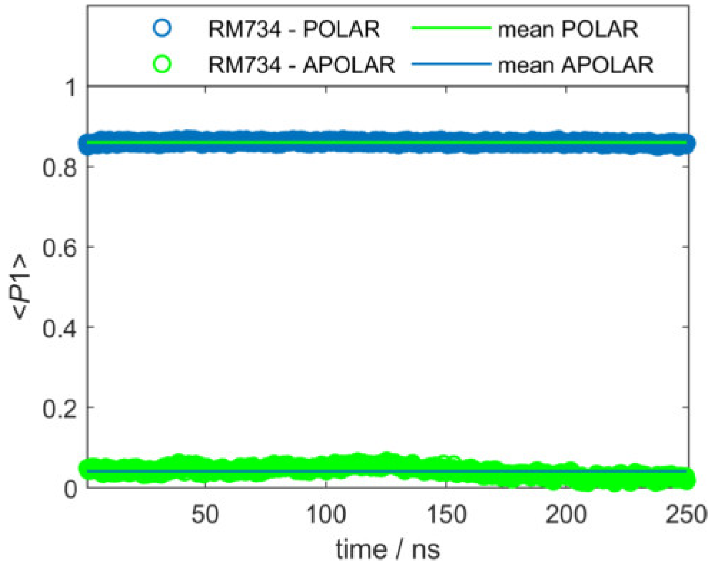

Supplementary Fig. 21: Plot of the <*P1*> order parameter as a function of simulation time for RM734 in the polar and apolar configurations at 400 K, and its mean value in both.

Supplementary References

1. Sebastián, N., Mandle, R. J., Petelin, A., Eremin, A. & Mertelj, A. Electrooptics of mm-scale polar domains in the ferroelectric splay nematic phase. *ArXiv210310215 Cond-Mat* (2021).
2. Havriliak, S. & Negami, S. A Complex Plane Analysis of alfa-Dispersions in Some Polymer Systems. *J Polym Sci C* **14**, 99–117 (1966).
3. Luigi Nordio, P., Rigatti, G. & Segre, U. Dielectric relaxation theory in nematic liquids. *Mol. Phys.* **25**, 129–136 (1973).
4. Sebastián, N. *et al.* Ferroelectric-Ferroelastic Phase Transition in a Nematic Liquid Crystal. *Phys. Rev. Lett.* **124**, 037801 (2020).
5. Mertelj, A. *et al.* Splay Nematic Phase. *Phys. Rev. X* **8**, 041025 (2018).
6. Mandle, R. J. & Mertelj, A. Orientational order in the splay nematic ground state. *Phys. Chem. Chem. Phys.* **21**, 18769–18772 (2019).
7. M. J. Frisch, G. W. Trucks, H. B. Schlegel, G. E. Scuseria, M. A. Robb, J. R. Cheeseman, G. Scalmani, V. Barone, G. A. Petersson, H. Nakatsuji, X. Li, M. Caricato, A. Marenich, J. Bloino, B. G. Janesko, R. Gomperts, B. Mennucci, H. P. Hratchian, J. V. Ortiz, A. F. Izmaylov, J. L. Sonnenberg, D. Williams-Young, F. Ding, F. Lipparini, F. Egidi, J. Goings, B. Peng, A. Petrone, T. Henderson, D. Ranasinghe, V. G. Zakrzewski, J. Gao, N. Rega, G. Zheng, W. Liang, M. Hada, M. Ehara, K. Toyota, R. Fukuda, J. Hasegawa, M. Ishida, T. Nakajima, Y.

Honda, O. Kitao, H. Nakai, T. Vreven, K. Throssell, J. A. Montgomery, Jr., J. E. Peralta, F. Ogliaro, M. Bearpark, J. J. Heyd, E. Brothers, K. N. Kudin, V. N. Staroverov, T. Keith, R. Kobayashi, J. Normand, K. Raghavachari, A. Rendell, J. C. Burant, S. S. Iyengar, J. Tomasi, M. Cossi, J. M. Millam, M. Klene, C. Adamo, R. Cammi, J. W. Ochterski, R. L. Martin, K. Morokuma, O. Farkas, J. B. Foresman, and D. J. Fox. *Gaussian 09, Revision D01*. (Gaussian, Inc., 2016).

8. Zhao, Y. & Truhlar, D. G. Density Functional for Spectroscopy: No Long-Range Self-Interaction Error, Good Performance for Rydberg and Charge-Transfer States, and Better Performance on Average than B3LYP for Ground States. *J. Phys. Chem. A* **110**, 13126–13130 (2006).
9. Grimme, S., Antony, J., Ehrlich, S. & Krieg, H. A consistent and accurate ab initio parametrization of density functional dispersion correction (DFT-D) for the 94 elements H-Pu. *J. Chem. Phys.* **132**, 154104 (2010).
10. Kendall, R. A., Dunning, T. H. & Harrison, R. J. Electron affinities of the first-row atoms revisited. Systematic basis sets and wave functions. *J. Chem. Phys.* **96**, 6796–6806 (1992).
