# Supplementary material for: On the molecular origins of the ferroelectric splay nematic phase
Source: Nat Commun. 2021 Aug 16;12:4962. doi: 10.1038/s41467-021-25231-0 (PMC8367997; doi:10.1038/s41467-021-25231-0)
Supplement: Supplementary file 3 — Description of Additional Supplementary Files [file 41467_2021_25231_MOESM3_ESM.pdf]

Description of Additional Supplementary Files

File name: Supplementary Movie 1
Description: Visualization of volume changes for RM734 in a capillary of 500 µm width and 50 µm. The sample is cooled from the isotropic phase while monitoring the position of the meniscus. Polarizers are slightly uncrossed and the position of the meniscus is taken by the image minimum intensity in the central section of the capillary. Transitions from isotropic to nematic and from nematic to polar splay nematic phase are clearly visible at 192 °C and 132 °C respectively.
